# The Relationship between Climbing Ability and Physiological Responses to Rock Climbing

**DOI:** 10.1155/2014/678387

**Published:** 2014-01-27

**Authors:** Jiří Baláš, Michaela Panáčková, Barbora Strejcová, Andrew J. Martin, Darryl J. Cochrane, Miloš Kaláb, Jan Kodejška, Nick Draper

**Affiliations:** ^1^Faculty of Physical Education and Sport, Charles University in Prague, 16252 Prague, Czech Republic; ^2^School of Sport & Exercise, Massey University, Palmerston North 4442, New Zealand; ^3^School of Sport & Physical Education, University of Canterbury, Christchurch, Canterbury 8140, New Zealand

## Abstract

*Aim*. The aim of this study was to examine the relationship between submaximal and maximal physiological responses to rock climbing for climbers of differing abilities. *Methods*. Twenty-six male climbers performed a submaximal climbing test on a known circuit at 90° (vertical) and 105° (15° overhanging) inclination and speed 25 movements*·*min^−1^. A maximal test was undertaken on a similar circuit at the same speed with inclination increasing by 10° for each successive 3 min stage. *Results*. Mean oxygen consumption and heart rate (HR) increased with wall inclination and climbers reached a mean (±SD) peak $\dot{\text{V}}{\text{O}}_{2}$ of 40.3 ± 3.5 mL*·*kg^−1^ *·*min^−1^ during the maximal test. Self-reported climbing ability was negatively correlated with $\dot{\text{V}}{\text{O}}_{2}$ and HR during the submaximal test at 90° ($\dot{\text{V}}{\text{O}}_{2}$, *r* = −0.82; HR, and *r* = −0.66) and at 105° ($\dot{\text{V}}{\text{O}}_{2}$, *r* = −0.84; HR, and *r* = −0.78) suggesting an increased exercise economy for climbers with a higher ability level. *Conclusion*. Findings from this study indicate that there is a relationship between wall inclination and the physiological demand of a climb. However, the increased technical ability and fitness of higher level climbers appears to an extent to offset the increased demand through improved exercise economy which in turn leads to an increased time to exhaustion and an improvement in performance.

## 1. Introduction

Interest in the physiology of sport climbing has grown among sport scientists over the past 25 years. Research studies have examined climbers' anthropometric, physiological, performance, and injury profiles [1–9] and focused on strength/endurance characteristics of forearm muscles [10–14]. Further research has shown that the physiological responses during climbing have varied with the length and style of the ascent [15, 16], the speed and direction of the movement [17, 18], the inclination and the surface of the climbing holds, and the overall difficulty of the ascent [1, 18–20].

Overall climbing difficulty is generally classified by a combination of factors such as wall inclination and the number of holds, as well as their size and shape. Since climbing speed is chosen by personal rhythm, except for competitive speed climbing, the inclination of a climb should be considered an important factor resulting in an increased physiological response. The increase in physiological responses with increasing inclination during submaximal climbing was first demonstrated in studies by Mermier et al. [19] and Watts and Drobish [20]. However, Mermier et al. (1997) did not indicate the speed during the ascents in three inclinations (90°, 106°, and 151°). Watts and Drobish (1998) stated that with increasing inclination there was a decrease in climbing rate. As the speed of the ascents was not determined in their study, the relationship between inclination and the physiological response evoked remains to be determined.

In a summary of seven climbing studies, Watts [8] indicated that after 80–100 s of climbing oxygen uptake ($\dot{\text{V}}{\text{O}}_{2}$) averaged 20–25 mL·kg^−1^·min^−1^ and peak oxygen uptake occurred at a point slightly over 30 mL·kg^−1^·min^−1^. In recent studies, de Geus et al. [17] and Draper et al. [16] reported peak values of  $\dot{\text{V}}{\text{O}}_{2}$ exceeding 40 mL·kg^−1^·min^−1^ underlining the role of the aerobic energy system when climbing near an individual's maximum. Additionally, a peak oxygen uptake of approximately 50 mL·kg^−1^·min^−1^ has been documented during a climbing test with increasing speed until exhaustion [21]. However, as España-Romero et al. stated that the specificity of their chosen protocol could be realised further if the difficulty of the route intensified gradually due to an increase in wall inclination rather than progressive changes in climbing rate/speed.

Discrepancies in physiological responses to climbing between studies may be due to differences between sample groups selected, but also possibly related to the adoption of self-paced climbing protocols. Therefore, it perhaps remains a point of contention as to whether reported climbing peak oxygen uptakes are related to climbing ability and physiological adaptation or to the climbing speed employed during ascent. Therefore, the aim of our study, with climbing speed held constant, was to examine the relationship between climbing ability and physiological responses to submaximal and maximal climbing.

## 2. Materials and Methods

### 2.1. Participants

Twenty-six male climbers with mean (±SD) age 26.8 ± 3.3 years; body mass 70.6 ± 6.2 kg; height 1.78 ± 0.07 m volunteered to participate in the study. The self-reported climbing ability of participants ranged from beginner to elite level. The climbers in this study had a self-reported red-point climbing ability from IV–X on UIAA scale (3–8b Sport; 5.4–5.13d YDS) (UIAA is Union Internationale des Associations d'Alpinisme; Sport = Sport or French grade system; YDS is Yosemite Decimal System). Previous research indicates that self-reported climbing ability assessment appears to provide a valid and reliable measure of performance [22]. The study received approval from the local ethics committee and written informed consent was obtained from all participants. All experimental procedures were conducted in accordance with the Declaration of Helsinki (1964).

### 2.2. Climbing Test

The climbing was undertaken on a 3 m high and 3 m wide bouldering wall that permitted progressive changes of inclination from vertical (90°) to overhanging profile (135°). Large mattresses placed on the floor under the wall enabled climbing without the need for harnesses or belaying equipment. The test started with submaximal climbing on a known circuit at 90° and 105° inclination at a speed of 25 movements·min^−1^. The speed was determined after prior trials and consultation with the climbers and enabled climbing at all inclinations without any limitation in technical execution of the climbing movements. Each movement was counted when a hand changed position from one hold to another, climbers individually moved their feet between holds as required. The circuit contained 15 climbing movements where the starting and the final hold were the same. The circuit contained upclimbing, traversing, and downclimbing. Each climber had to perform 5 circuits at 90° immediately followed by 5 circuits at 105° during submaximal climbing (5 circuits × 15 movements = 75 movements during 3 minutes at a speed of 25 movements·min^−1^). The speed of climbing was led by a digital metronome and controlled by the researcher for the duration of the circuit. The difficulty of the climbs at 90° and 105° were estimated as III and IV+ on the UIAA scale, respectively (3^+^ and 4 Sport; 5.4 and 5.5 YDS).

After submaximal climbing, all climbers received a 4-minute rest before undertaking the maximal test on a second known circuit. However, there was an exception, two climbers with the lowest climbing abilities did not recover after the submaximal test and were allowed to complete the maximal test 2 days later. The maximal test started for less advanced climbers (climbing ability <7 UIAA/6b Sport/5.10c YDS) at 95° inclination, for more advanced at 105° (climbing ability ≥7 UIAA/6b Sport/5.10c YDS), and after every 3 minutes the wall was inclined by 10° without any climbing interruption. The more advanced climbers started at a higher inclination so that the maximal test would not last too long and the results would not be affected by a decline in motivation. The test was finished by the fall of the climber due to the accumulated fatigue when the climber could not follow the given speed. When a fall occurred after a technical mistake, the climber could immediately continue with the test.

### 2.3. Treadmill Test

Maximal running performance was determined by a graded exercise test on a treadmill (Quasar, H/P/Cosmos, Germany). The test started with two submaximal speeds (10, 12 km·h^−1^) at 0% inclination lasting for 8 minutes (2 × 4 min) followed by a 4-minute rest period. The maximal test was performed at 5% constant inclination and at a starting speed of 12 km·h^−1^, which was increased every minute by 1 km·h^−1^ until voluntary exhaustion. All participants attained at least two of the following criteria at the end of the test: respiratory exchange ratio (RER) higher than 1.1, oxygen uptake plateau, and heart rate (HR) higher than 90% of age predicted-maximal HR (HR_max⁡_).

### 2.4. Respiratory and Heart Rate Analysis

Minute ventilation (${\dot{\text{V}}}_{\text{E}}$), oxygen uptake ($\dot{\text{V}}{\text{O}}_{2}$), and carbon dioxide production ($\dot{\text{V}}\text{C}{\text{O}}_{2}$) were measured during the climbing and treadmill tests by a portable breath-by-breath indirect calorimetry system (MetaMax 3B, Cortex Biophysic, Germany). The MetaMax 3B was secured onto the chest by a harness. Before each test, gas and volume calibration was performed according to manufacturer's guidelines. The volume calibration was performed using a known 3L syringe and gas calibration was performed with a known gas mixture of 15% O_2_ and 5% CO_2_. Data was averaged over 20 s intervals; the mean of the last minute from submaximal climbing and the highest values from the maximal test were taken into analysis. RER was computed by dividing measured CO_2_ by measured O_2_. HR was monitored by the MetaMax 3B using a polar heart transmitter belt (Polar Electro OY, Finland). Heart rate maximum (HR_max⁡_) was defined as the highest value attained during the test (recorded from 20 s averaged data).

### 2.5. Statistical Analysis

All variables demonstrated normality of distribution as assessed by one sample Kolmogorov-Smirnov goodness of fit testing. Descriptive statistics (means and SD) were used to characterize the physiological responses during climbing and treadmill tests. The relationship between climbing ability and cardiopulmonary variables was verified by Pearson product moment correlation. We considered the strength of the relationship (*R* ^2^) according to Ferguson [23] to be 0.2 minimum practical effect; 0.5 moderate effect; 0.8 strong effect. To calculate the climbing relative intensity, the individual climbing maximal values were related to corresponding values from the treadmill test. An *α* level of 0.05 was set to accept significance for each inferential test. All statistical analyses were performed using statistical software SPSS for Windows Version 19 (Chicago, IL, USA).

## 3. Results

**Figure 1:**
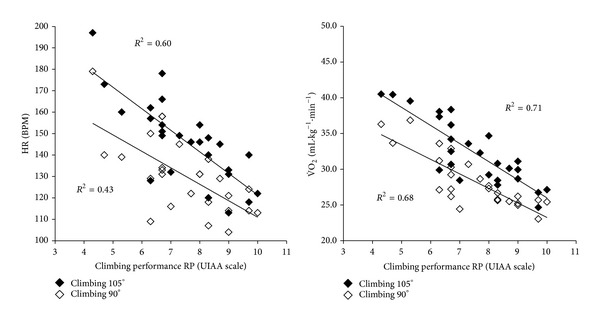
The relationship between climbing ability and oxygen uptake ($\dot{\text{V}}{\text{O}}_{2}$) and heart rate (HR) during submaximal climbing test.

Descriptive data for the climbers are presented in Table 1. As can be seen from this table, trends were as expected, with mean HR, ${\dot{\text{V}}}_{\text{E}}$, $\dot{\text{V}}{\text{O}}_{2}$, and RER rising with increased wall inclination. The mean climbing specific oxygen consumption ($\dot{\text{V}}{\text{O}}_{2\;\text{climbing-peak}}$) was 40.3 ± 3.5 mL·kg^−1^·min^−1^, which represented ~68% of the treadmill $\dot{\text{V}}{\text{O}}_{2\max}$. The nature of the relationship of climbing ability with oxygen consumption and HR, along with meaningfulness of each relationship, is shown in Figure 1. There was a significant negative correlation between climbing ability and $\dot{\text{V}}{\text{O}}_{2}$ at 90° and at 105° (*r* = −0.82, *P* < 0.05; *r* = −0.84, *P* < 0.05) and HR (*r* = −0.43, *P* < 0.05; *r* = −0.78, *P* < 0.05), respectively. These results suggest that the higher the ability of the climber the lower the physiological response ($\dot{\text{V}}{\text{O}}_{2}$ and HR) to climbing at a submaximal intensity. Interestingly, climbing ability most strongly predicted the level of wall inclination attained by each climber at the moment of exhaustion (*r* = 0.89, *R* ^2^ = 0.79).

## 4. Discussion

The main aim of our study was to determine physiological responses to climbing with progressive inclination during submaximal and maximal climbing tests and to examine their relationship with climbing ability. The selected participants represented all levels of climbing abilities from beginners to elite level climbers. To the best of our knowledge, this is the first study to assess the effect of inclination where the speed and the route were held constant. During the submaximal test, climbers with higher ability demonstrated lower $\dot{\text{V}}{\text{O}}_{2}$ and HR and as a consequence a greater economy of movement, which is consistent with the findings of previous research [24, 25]. The mean $\dot{\text{V}}{\text{O}}_{2}$ for more advanced climbers was ~26 and ~30 mL·kg^−1^·min^−1^, and for lower grade climbers the mean was ~31 and ~36 mL·kg^−1^·min^−1^ at 90° and 105°, respectively, indicating that the more advanced climbers were able to expend approximately one fifth of the energy less than expended by the lower grade climbers.

Mermier et al. [19] evaluated physiological responses during self-paced climbing at three inclinations (90°, 106°, and 151°), where the first two angles are comparable to our study. In experienced climbers (climbing ability not defined), the authors reported a $\dot{\text{V}}{\text{O}}_{2}$ of 20.7 ± 8.1 mL·kg^−1^·min^−1^ at 90° and 21.9 ± 5.3 mL·kg^−1^·min^−1^ at 106°, which is lower than in the more advanced climbers in our study. The lower $\dot{\text{V}}{\text{O}}_{2}$ in the Mermier et al. [19] study may be explained by the self-selected speed and probably slower rate of ascent or by the fact that climbers were top-roping up and down, where the down climbing would have been much easier than the ascents.

Watts and Drobish [20] assessed the effect of five inclinations (80°, 86°, 91°, 96°, and 102°) on a special climbing treadmill in novice climbers (climbing ability not defined). The authors found similar $\dot{\text{V}}{\text{O}}_{2}$ at all angles, ranging from 29.7 to 31.5 mL·kg^−1^·min^−1^ and increasing mean HR rising from 156 to 171 beats·min^−1^. The self-selected climbing speed decreased with higher angle from 89.9 m to 27.0 m over 4 min. The authors stated that some combined effect of climbing difficulty and rate of ascent balanced the overall energy requirement such that $\dot{\text{V}}{\text{O}}_{2}$ remained constant. The increasing HR despite similar $\dot{\text{V}}{\text{O}}_{2}$ was explained by greater stress on the upper body and increased sympathetic drive during arm exercise. Our results confirmed a significant effect of the inclination on $\dot{\text{V}}{\text{O}}_{2}$, ${\dot{\text{V}}}_{\text{E}}$, RER, and HR, when climbing speed is held constant.

During the maximal climbing test, the attained $\dot{\text{V}}{\text{O}}_{2}$ corresponded to the peak values of de Geus et al. [17] and Draper et al. [16]. de Geus et al. [17] reported $\dot{\text{V}}{\text{O}}_{2}$ of 41.3 ± 4.9 mL·kg^−1^·min^−1^ during top-rope climbing and bouldering at self-selected speed and near-maximal difficulty (79 ± 11% of the running maximum, 52.2 ± 5.1 mL·kg^−1^·min^−1^). The percentage of climbing $\dot{\text{V}}{\text{O}}_{2}$ to the running maximum was higher than our value (68 ± 7%), probably due to the higher aerobic fitness of our climbers. This perhaps suggests that the $\dot{\text{V}}{\text{O}}_{2\;\text{climbing-peak}}$ is not influenced by the level of aerobic fitness. However, climbers with low aerobic fitness (less than 45 mL·kg^−1^·min^−1^) may be limited during climbing to exhaustion by the cardiorespiratory system. Draper et al. [16] found a peak $\dot{\text{V}}{\text{O}}_{2}$ during top rope climbing of 38.3 ± 5.9 mL·kg^−1^·min^−1^ and lead climbing of 40.9 ± 6.6 mL·kg^−1^·min^−1^ at a level of difficulty “that failure to complete the climb was a realistic possibility for all participants” [16]. These authors found the speed in lead climbing significantly slower than in top rope climbing, 3.1 min versus 1.3 min, respectively, for a 9.38 m high climb. It is noted that a plateau around 40 mL·kg^−1^·min^−1^ appeared during climbing at near-maximal difficulty and is independent of self-selected speed. In contrast, Magalhães et al. [26] reported an oxygen uptake during self-paced lead climbing of near-maximal difficulty of 33.4 ± 2.1 mL·kg^−1^·min^−1^ which represented 61% of running maximum (54.5 ± 2.1 mL·kg^−1^·min^−1^). The discrepancy may be explained by the methodology of the climbing protocol, which could have included a short rest from lowering the climbers from the top anchor, where authors have used mean $\dot{\text{V}}{\text{O}}_{2}$ for the whole climb instead of peak values.

Although the role of self-paced speed does not apparently have an effect on peak $\dot{\text{V}}{\text{O}}_{2}$ during climbing at near maximal difficulty, the effect of a given speed may have a substantial role [18, 21]. Booth et al. [18] used increasing speed, instead of inclination, on a motorized climbing treadwall to determine $\dot{\text{V}}{\text{O}}_{2\text{peak}}$ where novice climbers achieved 43.8 ± 2.2 mL·kg^−1^·min^−1^. The same protocol was used by España-Romero et al. [21] with highly experienced climbers and the peak $\dot{\text{V}}{\text{O}}_{2}$ ranged from 49.2 ± 3.5 mL·kg^−1^·min^−1^ for women to 53.6 ± 3.7 mL·kg^−1^·min^−1^ for men. The high $\dot{\text{V}}{\text{O}}_{2\text{peak}}$ in the España-Romero et al. study [21] can be explained by a longer time to exhaustion and higher climbing ability compared to Booth et al.'s study [18]. España-Romero et al. [21] found the time to exhaustion significant to the climbing performance but not the value of $\dot{\text{V}}{\text{O}}_{2\text{peak}}$ (Spearmen correlation coefficient for both sexes, *ρ* = 0.32). However, if the sample of climbers was more heterogeneous in climbing abilities, we might expect a stronger relationship. Neither the study of Booth et al. [18] or the study of España-Romero et al. [21] evaluated the nonspecific $\dot{\text{V}}{\text{O}}_{2\text{peak}}$ on treadmill or cycle ergometer. Thus the relationship of climbing specific and nonspecific $\dot{\text{V}}{\text{O}}_{2\text{peak}}$ cannot be evaluated.

Studies by Booth et al. [18] and España-Romero et al. [21] suggest that a climbing protocol with increasing speed elicits a higher specific $\dot{\text{V}}{\text{O}}_{2}$ than climbing protocols with increasing difficulty (inclination, holds configuration). There are several explanations. For example, overhanging climbing involves a considerable degree of time spent in static contraction of the upper limbs and upper body which can deteriorate the pulmonary ventilation and therefore transport of oxygen. In that study, the ${\dot{\text{V}}}_{\text{Emax}}$ during climbing (74.9 ± 10.1 L·min^−1^, 53% of the running maximum) was substantially lower than the ${\dot{\text{V}}}_{\text{Emax}}$ [21] in the climbing protocol with increasing speed (138.7 ± 25.6 L·min^−1^). The difference perhaps reveals the effect of lower speed and inclination on the pulmonary ventilation volume when climbing to exhaustion.

There was an interesting finding in ${\dot{\text{V}}}_{\text{E}}/\dot{\text{V}}{\text{O}}_{2}$ ratio. A moderate relationship (*r* = 0.61, *R* ^2^ = 0.38) was found between ${\dot{\text{V}}}_{\text{E}}/\dot{\text{V}}{\text{O}}_{2}$ in the maximal climbing test and the treadmill test. In addition, climbers with higher climbing ability tended to achieve higher ${\dot{\text{V}}}_{\text{E}}/\dot{\text{V}}{\text{O}}_{2}$ ratio (hyperventilation) than lower level climbers and attained a higher RER. The following questions arise, are advanced climbers able to exceed their ventilatory anaerobic threshold by having a stronger upper body or are less advanced climbers limited in their breathing rate during climbing due to their weaker upper body strength? Often, climbers are found not breathing during the difficult moves in the ascent. However, the coupling between respiration and locomotion could provide favourable conditions for improvement in athletic performance [27, 28]. Further study is required to examine if induced breathing during overhanging climbing can enhance climbing performance.

In conclusion, we found a significant relationship between climbing ability and the physiological response to submaximal climbing. Our data suggest that the $\dot{\text{V}}{\text{O}}_{2}$ during submaximal climbing perhaps provides a useful parameter with which to estimate climbing economy. There was a strong correlation between climbing ability and the climbing test with progressive inclination and a constant speed of 25 movements·min^−1^ until exhaustion. This suggests that this test may represent a suitable method through which to assess the aerobic component of climbing performance.

## Figures and Tables

**Table 1:** Mean (±SD) oxygen uptake (${\dot{\text{V}}\text{O}}_{\text{2}}$), heart rate (HR), minute ventilation (${\dot{\text{V}}}_{\text{E}}$), and respiratory exchange ratio (RER) in the submaximal test.

	Submaximal climbing test (90°)	Submaximal climbing test (105°)	Maximal climbing test	Maximal treadmill test	% of treadmill maximum
${\dot{\text{V}}\text{O}}_{\text{2}}$ (mL·kg^−1^·min^−1^)	28.5 ± 3.6	32.4 ± 4.3	40.3 ± 3.5	59.7 ± 5.1	0.68 ± 0.07
HR (beats·min^−1^)	130 ± 17	146 ± 19	178 ± 11	193 ± 8	0.92 ± 0.04
${\dot{\text{V}}}_{\text{E}}$ (L·min^−1^)	41.3 ± 6.9	49.7 ± 11.5	74.9 ± 10.1	139.3 ± 11.9	0.54 ± 0.09
RER	0.79 ± 0.06	0.86 ± 0.06	0.98 ± 0.07	1.16 ± 0.04	0.85 ± 0.07
Time (min:s)			6:43 ± 2:35	5:11 ± 1:04	
